# ﻿Dryopteris×subdiffracta (Dryopteridaceae), a new natural hybrid fern from Guangxi, China

**DOI:** 10.3897/phytokeys.250.119803

**Published:** 2024-12-18

**Authors:** Hong-Jin Wei, Zheng-Yu Zuo

**Affiliations:** 1 Eastern China Conservation Centre for Wild Endangered Plant Resources, Shanghai Chenshan Botanical Garden, Shanghai, 201602, China Shanghai Chenshan Botanical Garden Shanghai China; 2 Germplasm Bank of Wild Species, Kunming Institute of Botany, Chinese Academy of Sciences, Kunming, Yunnan, 650201, China Kunming Institute of Botany, Chinese Academy of Sciences Kunming China

**Keywords:** *Dryopteris* sect. *Acrorumohra*, hybridization, molecular phylogeny, nuclear gene *AK1*, plastome

## Abstract

A new natural hybrid fern, Dryopteris×subdiffracta (Dryopteridaceae), is reported from Guangxi, China. Molecular phylogenetic analysis based on DNA sequences from the low-copy nuclear marker *Ak1* and plastid genome revealed respectively that *D.polita* and *D.diffracta* are parents of the new hybrid, with *D.polita* as the maternal parent. Cytometric analysis of the nuclear DNA content indicated that *D.×subdiffracta* might be a diploid hybrid. Morphologically, *D.×subdiffracta* shares a high degree of similarity with *D.×subreflexipinna* from Taiwan, especially in zigzag-shaped rachis and deflexed pinna stalks. However, *D.×subdiffracta* is distinguishable in the degree of lamina division and shapes of lamina and pinnulets. A comprehensive taxonomic description accompanied by line drawings are provided.

## ﻿Introduction

*Dryopteris* Adans. (1763: 20) is the third largest genus of ferns worldwide, comprising about 400 species (Wu et al. 2013). In China, the newly updated fern checklist includes approximately 188 *Dryopteris* taxa (180 species), making it the second largest fern genus after *Polystichum* Roth (Liu et al. 2023). Recent studies have shown that many *Dryopteris* taxa are of hybrid origin (e.g., Juslén et al. 2011; Hori et al. 2014, 2018, 2021; Sessa et al. 2015; Zuo et al. 2021; Wei et al. 2024). The majority of natural interspecific fern hybrids could be detected initially by their abnormal spores and intermediate morphological characteristics between two distinct ferns in the field (Wagner and Chen 1965; Barrington 1990).

In 2018, a suspicious fern was collected in the Dayaoshan Mountains, the largest mountain in the east-center of Guangxi, China. This fern bears a striking resemblance to *Dryopteris×subreflexipinna* M.Ogata (1935), an endemic fern to Taiwan which was assumed to be a hybrid resulting from a cross between *D.diffracta* (Baker) C.Chr. and *D.hasseltii* (Blume) C.Chr. by Moore (2000), and later verified by Chang et al. (2009). However, the former was always found growing together with *D.diffracta* and *D.polita* Rosenst. In addition, *D.hasseltii* has never been recorded in the Dayaoshan Mountains. The closest recorded site of collection of *D.hasseltii* is approximately 93 km away as the crow flies based on our data. These suggested that the newly discovered fern is probably not D.×subreflexipinna, but may rather result from a different parental combination. We named this presumptive new hybrid *D.×subdiffracta* herein, and try to determine its parentage through morphology, palynology, cytology and molecular biology.

## ﻿Materials and methods

### ﻿Taxon sampling and morphological comparison

Samples included *Dryopteris×subdiffracta* and its two associated species (i.e., supposed parents), *D.diffracta* and *D.polita*, collected at the same location. Additionally, *D.hasseltii*, the maternal progenitor of D.×subreflexipinna (Chang et al. 2009; Zhang et al. 2012), and other species demonstrated to be closely related to *D.polita* in D.sect.Acrorumohra (Li and Lu 2006; Zhang et al. 2012) were also sampled. Morphological traits of *D.×subdiffracta* and D.×subreflexipinna were based on specimens in Shanghai Chenshan Herbarium (CSH), Herbarium of Guangxi Institute of Botany (IBK) and Herbarium of Kunming Institute of Botany (KUN), as well as digital images of specimens in network databases, such as CVH (https://www.cvh.ac.cn/), Digital Taiwan (https://digitalarchives.tw/), Plants of TAIWAN (https://tai2.ntu.edu.tw/index.php) and GBIF (https://www.gbif.org/).

### ﻿DNA extraction, sequencing, and plastome assembly

Plastid DNA was used as the maternal inherited marker and *AK1* as a biparentally inherited low-copy nuclear marker. Total DNA was extracted using an improved extraction CTAB method (Doyle and Doyle 1987) from 20 mg of silica gel-dried leaf material. Library construction, Illumina sequencing and plasmid DNA assembly followed Zuo et al. (2021). Primer design and reaction protocols for low-copy nuclear *AK1* gene followed Hori et al. (2021). GenBank accession numbers for the six new samples are listed in Table 1.

**Table 1.** Taxon, voucher specimen information, GenBank accession numbers, nuclear DNA content, and the number of spores per sporangium of *Dryopteris* samples used in this study.

Taxon	Specimen	Locality	Plastome	*AK1* copies	pg/2C	Spores	Ploidy
*D.diffracta*	*JSL6261*	Jinxiu, Guangxi, China	PQ167731	PP277076			
*D.diffracta*	*Zuo1817*	Gongshan, Yunnan, China	OQ649848	PP277077	19.49 ± 0.54	64	2×
*D.diffracta*	*Zuo5590*	Jinxiu, Guangxi, China	PQ167732	PP277078	20.20 ± 0.71	64	2×
*D.hasseltii*	*DLJ2019235*	Gongshan, Yunnan, China	OQ649872	PP277079	20.08 ± 0.15	64	2×
*D.polita*	*JSL6338*	Jinxiu, Guangxi, China	PQ167733	PP277082			
*D.polita*	*Zuo5577*	Jinxiu, Guangxi, China	PQ167734	PP277083	14.65 ± 0.32	64	2×
D.×subdiffracta	*JSL6337*	Jinxiu, Guangxi, China	PQ167735	PP277084; PP277085			
D.×subdiffracta	*JSL6341*	Jinxiu, Guangxi, China	PQ167736	PP277086; PP277087			
D.×subdiffracta	*Zuo5589*	Jinxiu, Guangxi, China	PQ167737	PP277088; PP277089	16.82 ± 0.17		2×

### ﻿Data matrices and phylogenetic analyses

Two matrices were constructed for the analyses. The first matrix (plastid matrix) was comprised of nine entire chloroplast genome using Geneious 9.1.4 (Kearse et al. 2012), along with additional thirteen plastid regions (including *rbcL*, *trnL-F*, and *rps4-trnS*) obtained from GenBank. The second matrix (*AK1* matrix) included 11 nuclear *AK1* sequences extracted from our nine samples and another ten *AK1* sequences downloaded from GenBank. Alignment and correction of the matrices were performed using MAFFT v.7.017 (Katoh et al. 2002) and Geneious 9.1.4 (Kearse et al. 2012). Maximum likelihood (ML) analysis utilizing IQ-TREE 1.6.12 (Nguyen et al. 2015) was conducted with the GTR+R6 model and 1000 ultrafast bootstrap replicates. Bayesian inference (BI) analysis using MrBayes 3.2.6 (Ronquist et al. 2012) involved ten million generations with sampling every 1000 generations, employing four Markov chain Monte Carlo (MCMC) runs. The first 20% of trees were discarded as burn-in.

### ﻿Estimation of ploidy level and reproductive mode

Flow cytometry was employed to measure the nuclear DNA content (2C value) of individual cells extracted from fresh leaves (Hori et al. 2021). *Zeamays* L. was used as an internal standard. Ploidy level was inferred by comparison of observed nuclear content among samples and with previous reports. The number of spores in three complete sporangia per sample was counted under a small microscope (Yuantu 100×, China). For most of leptosporangiate ferns, sporangia with 64 and 32 spores, respectively, indicate sexual and apomictic reproduction (Manton 1950; Lovis 1977; Walker 1979; Grusz 2016).

## ﻿Results and discussion

### ﻿Morphological comparison

Morphological differences (marked with asterisk “*”) and similarities between Dryopteris×subdiffracta and D.×subreflexipinna are listed in Table 2. Dryopteris×subdiffracta shares high similarities with D.×subreflexipinna in the color and form of scales, pinna shape, degree of sinuosity of rachis, and angle between rachis and pinna stalks. Their zigzag rachis and deflexed pinna stalks are distinctly derived from *D.diffracta*, the only *Dryopteris* species with these characters in China, suggesting that both taxa might share at least an identical parent. However, there are many differences between D.×subdiffracta and D.×subreflexipinna. The most important difference lies in the shape of pinnulets, sometimes including pinnules. The pinnulet of D.×subdiffracta has a blunt or acute apex and a nearly symmetric base, its basalmost pair of lobes are nearly equal in size. In contrast, D.×subreflexipinna has a round or obtuse apex and an asymmetric base, with the spreading basalmost acroscopic pinnulet or lobe obviously larger than the ascending basiscopic one. This trait seemingly originated from one of its parents with the same characters, *D.hasseltii*. In terms of morphological intermediacy of hybrids, *D.diffracta* is a quadripinnate species which occasionally has one or two nearly free lobes on the base of some tertiary pinnules, whereas D.×subdiffracta is a tripinnate-pinnatifid plant with at most one or two free lobes at the bases of larger pinnulets, on the same level in lamina division as *D.hasseltii* rather than intermediate between the latter and *D.diffracta*. *Dryopterispolita* has bipinnate-pinnatipartite fronds, and in theory, is more likely the other parent of D.×subdiffracta, which also explains why D.×subdiffracta often has triangular laminae, like those of *D.polita* and *D.diffracta*. In contrast, D.×subreflexipinna generally has ovate-oblong laminae and shares the same lamina shape with its maternal parent *D.hasseltii*.

**Table 2.** Morphological comparison between Dryopteris×subdiffracta and D.×subreflexipinna.

Characters		D.×subdiffracta	D.×subreflexipinna
Scales	color	brown	brown
	shape	lanceolate	lanceolate
	margin	entire	entire
Frond length*		42–99 cm	65–123 cm
Lamina	division*	tripinnate to tripinnate-pinnatisect	4-pinnate
	shape*	triangular or ovate-triangular	ovate-oblong or ovate
	size*	22–44 × 15–34 cm	25–66 × 22–50 cm
	base	broadly cuneate* or rounded	rounded
Rachis form		slightly zigzag	slightly zigzag
Shape of lowest pinna		deltoid	deltoid
Included angle between rachis and deflexed pinna stalk		70–85°	70–85°
Size of basal basiscopic pinnule on lowest pinna*		36–90 × 17–37 mm	45–180 × 22–80 mm
Middle pinnules of pinnae	shape	deltoid-lanceolate	oblong-lanceolate* or deltoid-lanceolate.
	base*	nearly symmetrical; broadly cuneate acroscopically, broadly cuneate to cuneate basiscopically.	asymmetrical; truncate acroscopically, broadly cuneate to cuneate basiscopically.
	apex	shortly acuminate	shortly acuminate, acute or obtuse*
Pinnulets nonadjacent to costae	shape*	ovate to ovate-oblong	rhombic-ovate
	base*	cuneate to rounded-cuneate, nearly symmetrical	rounded-cuneate, asymmetrical
	apex	blunt* or obtuse	rounded* or obtuse
Relative size of proximal pair of pinnulets)*		acroscopic one nearly as large as or slightly larger than basiscopic one	acroscopic one significantly larger than basiscopic one
Relative size of proximal pair of ultimate pinnules (or lobes)*		acroscopic one nearly as large as or slightly larger than basiscopic one	acroscopic one significantly larger than basiscopic one

### ﻿Phylogenetic analyses

Phylogenetic analysis of the plastid matrix showed that three samples of *Dryopteris×subdiffracta* were fully supported to nest in the clade of *D.polita* (Fig. 2A). Meanwhile, three samples of *D.×subreflexipinna* were fully supported to nest in the clade of *D.hasseltii*. All three samples of *D.×subdiffracta* were found with two copies of *AK1*. Copy 1 was the same as *D.polita*, while copy 2 was the same as *D.diffracta* (Fig. 2B). This supports the hypothesis that D.×subdiffracta originated from hybridization between *D.polita* and *D.diffracta*.

### ﻿Estimation of ploidy level and reproductive mode

The mean DNA contents of D.×subdiffracta and related species are presented in Table 1. *Dryopterispolita* displayed the lowest value (14.65 ± 0.32 pg), whereas *D.diffracta* and *D.hasseltii* exhibited significantly higher values (approximately 20 pg), but still less than twice that of *D.polita*. *Dryopterisdiffracta* occurring in Taiwan had been reported to be tetraploid (Tsai and Shieh 1975, 1985; Chang et al. 2009), however, our samples from Guangxi possessed approximately 80 chromosomes per cell (data not shown), indicating that *D.diffracta* is highly likely diploid. *Dryopterishasseltii*, in our study, shared the similar DNA contents with *D.diffracta*, and so it was probably also diploid, which was in accord with some previous researches (e.g., Shimura et al. 1982; Kato and Nakato 1999). We also infer that *D.polita* is diploid.

**Figure 1.**
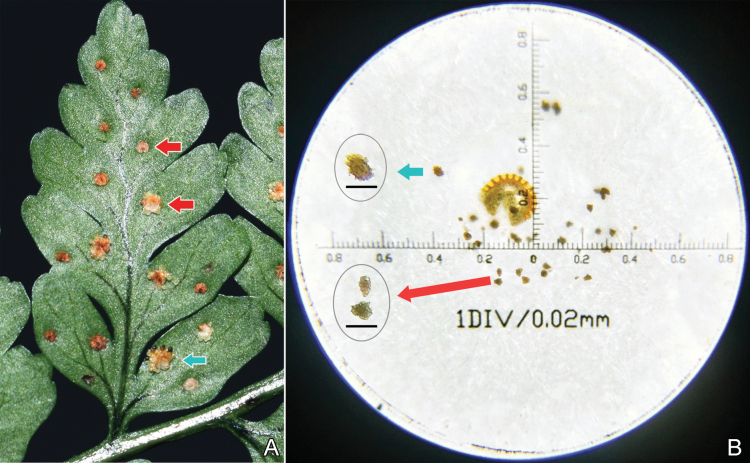
Dryopteris×subdiffracta**A** pinnules showing fertile sori (blue arrow) and infertile sori (red arrows) **B** sporangium showing mostly misshapen spores (red arrow) and a few normal spores (blue arrow), insets (circles) show detail. Photographed by Zheng-Yu Zuo. Scale bars: (in circles) 0.05 mm.

All the samples of *D.diffracta*, *D.hasseltii* and *D.polita* were observed with 64 spores per sporangium and considered to be sexual. The DNA content of samples of *D.×subdiffracta* was 16.82 ± 0.17 pg, and these samples were also estimated as being diploid. Most of sporangia of *D.×subdiffracta* were abortive. Some sporangia had a few normal spores occasionally, but most of these spores were misshapen (Fig. 1), which suggested that *D.×subdiffracta* is most likely sterile.

**Figure 2.**
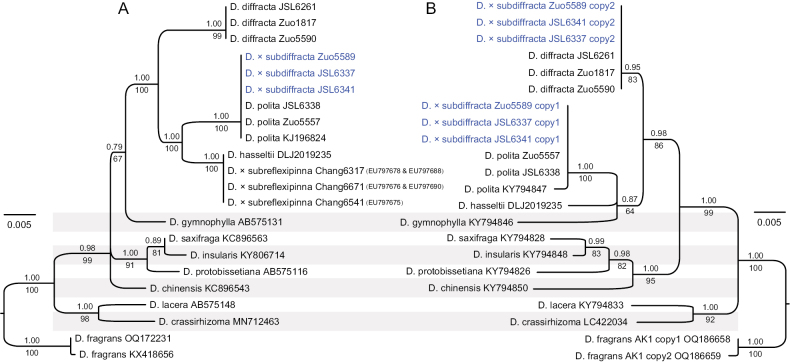
Maximum likelihood phylogram of Dryopterissect.Acrorumohra based on (**A**) plastid matrix and (**B**) nuclear *AK1* gene sequences. Support values, including bootstrap support values (BS) and Bayesian confidence values (PP), are depicted along the branches, with PP over the branches and BS under the branches. The NCBI accession numbers of sequences obtained from GenBank are given after the taxon names.

Previous studies reported that Dryopteris×subreflexipinna is a pentaploid hybrid produced from hybridization between diploid sexual *D.hasseltii* and tetraploid apogamous *D.diffracta* (Chang et al. 2009). Despite sharing some morphological similarities with *D.subreflexipinna*, our results indicate that D.×subdiffracta is a sterile diploid hybrid originating from the hybridization between two diploid sexual species, *D.polita* and *D.diffracta*.

### ﻿Taxonomy treatment

#### ﻿ Dryopteris × subdiffracta

Taxon classificationPlantaePolypodialesDryopteridaceae

H.J.Wei & Z.Y.Zuo nothosp. nov.

86BA6F4B-6072-5653-9EF2-699950854790

urn:lsid:ipni.org:names:77353653-1

Figs 3 , 4 , 5C, D

≡ D.diffracta (Baker) C. Chr. × D.polita Rosenst.

##### Type.

China • Guangxi: Jinxiu county, Mt. Shengtangshan, in broad-leaved forest, 23°58'11"N, 116°6'54"E, elev. 1156 m, 9 May 2018, *She-Lang Jin, Jing Liu, Qi-MingTang & Xu Yan JSL6337* (holotype: CSH0200999!; isotypes: CSH!, IBK!, KUN!).

##### Diagnosis.

Dryopteris×subdiffracta is similar to D.×subreflexipinna in having slightly flexuous rachis and deflexed pinna stalks, but the former has a tripinnate to tripinnate-pinnatisect frond, narrowly ovate or oblong pinnulets with obtuse apex and nearly symmetric base, while the latter has 4-pinnate frond, ovate pinnulets with round or obtuse-rounded apex and asymmetric base.

**Figure 3.**
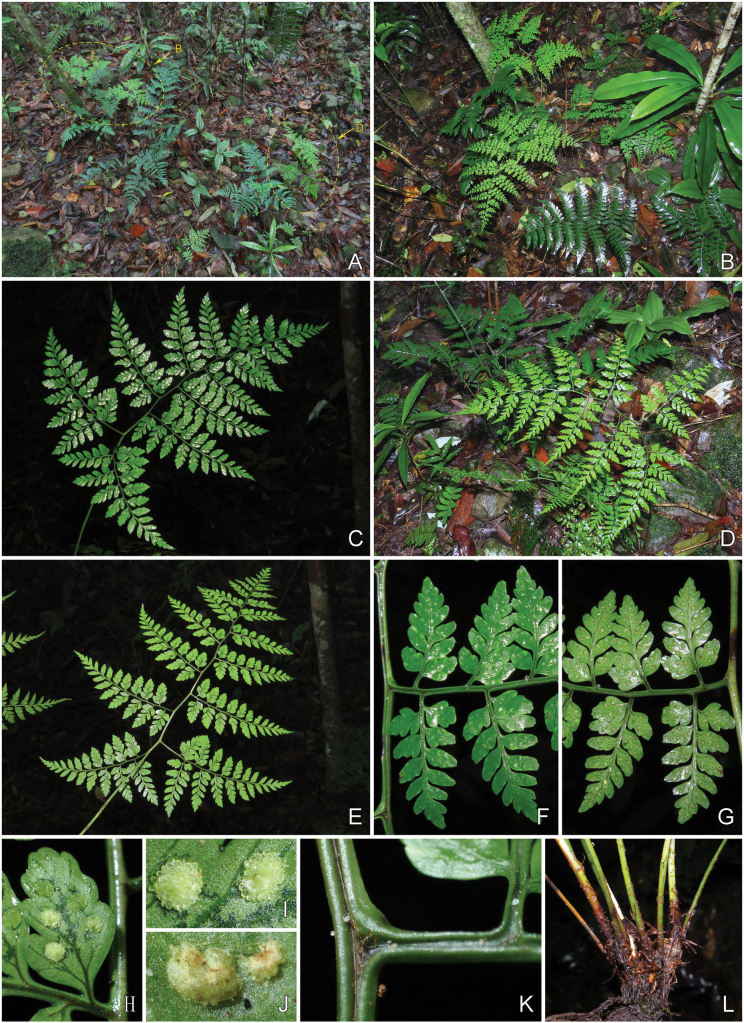
Dryopteris×subdiffracta**A** habitat showing the plant growing together with parents (arrows inset) **B***Dryopterisdiffracta* and *D.polita***C** adaxial view of lamina **D** habit **E** abaxial view of lamina **F** adaxial view of lower portion of middle pinna with portion of rachis **G** adaxial view of lower portion of middle pinna with portion of rachis **H** ultimate pinnule **I** sori **J** indusium **K** adaxial view of portion of rachis with lower portion of costae **L** rhizome with lower portion of stipes. Photographed by Hong-Jin Wei.

##### Description.

***Rhizome*** erect, densely covered with brown scales at apex. ***Frond*** (42–)50–84 cm; stipe stramineous, (23–)26–56 cm, (1–)1.5–3 mm in diam. at middle part, sparsely covered with scale at base, upwards glabrous, grooved adaxially; scales lanceolate, ca. 16 × 2.4 mm, brown, entire; ***lamina*** 3-pinnate to nearly 4-pinnate, deltoid to ovate-deltoid, (22–)27–43 × (15–)25–34 cm, base round or broadly cuneate, apex acuminate, rachis somewhat flexuous; ***pinnae*** 8–12 pairs, triangular-lanceolate, slightly falcate, alternate, lowest pair sometimes opposite to nearly opposite, stalked, stalks of lower pinnae slightly deflexed, bases forming an angle of ca. 70–85° with rachis, upswept distally, 2–6.5 cm apart from each other, stalks of middle pinnae spreading; ***lowest pinna*** largest, deltoid, (9–)12–21 × (6–)7–13 cm, base broadly cuneate or truncate, apex acuminate, stalk (1.5–)2–4 cm; ***pinnules*** 8–12 pairs, often anadromous, 1- or 2-pinnate, alternate, spreading, triangular-lanceolate, apex shortly acuminate, proximal pairs shortly stalked, bases broadly cuneate or shallowly cordate, nearly symmetrical, distal pairs of pinnules nearly sessile, bases asymmetrical, acroscopically broadly cuneate, basiscopically cuneate, basal acroscopic pinnule of pinna as large as or slightly smaller than adjacent ones, basal basiscopic pinnule on lowest pinna largest, (3.6–)5.5–7.5(–9) × (1.7–)2–3.7 cm, shortly acuminate, stalklet (1.5–)2–4 mm; ***pinnulets*** 6–10 pairs, often anadromous, proximal 2–5 pairs free, alternate, spreading to ascending, ovate to ovate-oblong, basal acroscopic one as large as or slightly larger than basiscopic one, bases of proximal pair rounded-cuneate, nearly symmetrical, bases of others rounded-cuneate to cuneate acroscopically, cuneate to narrowly cuneate basiscopically, apex blunt, acute to subacute, with 1 or 2 short obtuse teeth, larger pinnules (9.5–)11–18 × (5.5–)6.5–11 mm, stalklet 0.5–2 mm, pinnatifid or pinnatisect at base, with 1 or 2 free lobes proximally; ***lobes*** 1–3 pairs, ovate, narrowly ovate or oblong, ascending, margin undulant to entire, base narrowly cuneate, apex obtuse, entire or with 1 or 2 short obtuse teeth, larger lobes 3–6 × 2–3 mm; upper pinnae gradually reduced, spreading to oblique; ***lamina*** herbaceous, green on adaxial surface and light green on abaxial surface when dry, both surfaces glabrous, rachis and rachillae of every order stramineous, nearly glabrous expect for several hair-like scales on midribs and rachillae on abaxial surface, grooved adaxially; ***veins*** indistinct on adaxial surface, visible on abaxial surface, pinnate on pinnulets, veinlets forked or simple on lobes, not reaching margin; ***sori*** medial or slightly nearer to margin than to costa, terminal on veinlets, 1–3 pairs on each ultimate lobes; ***indusium*** brown, papery, entire, fugacious.

**Figure 4.**
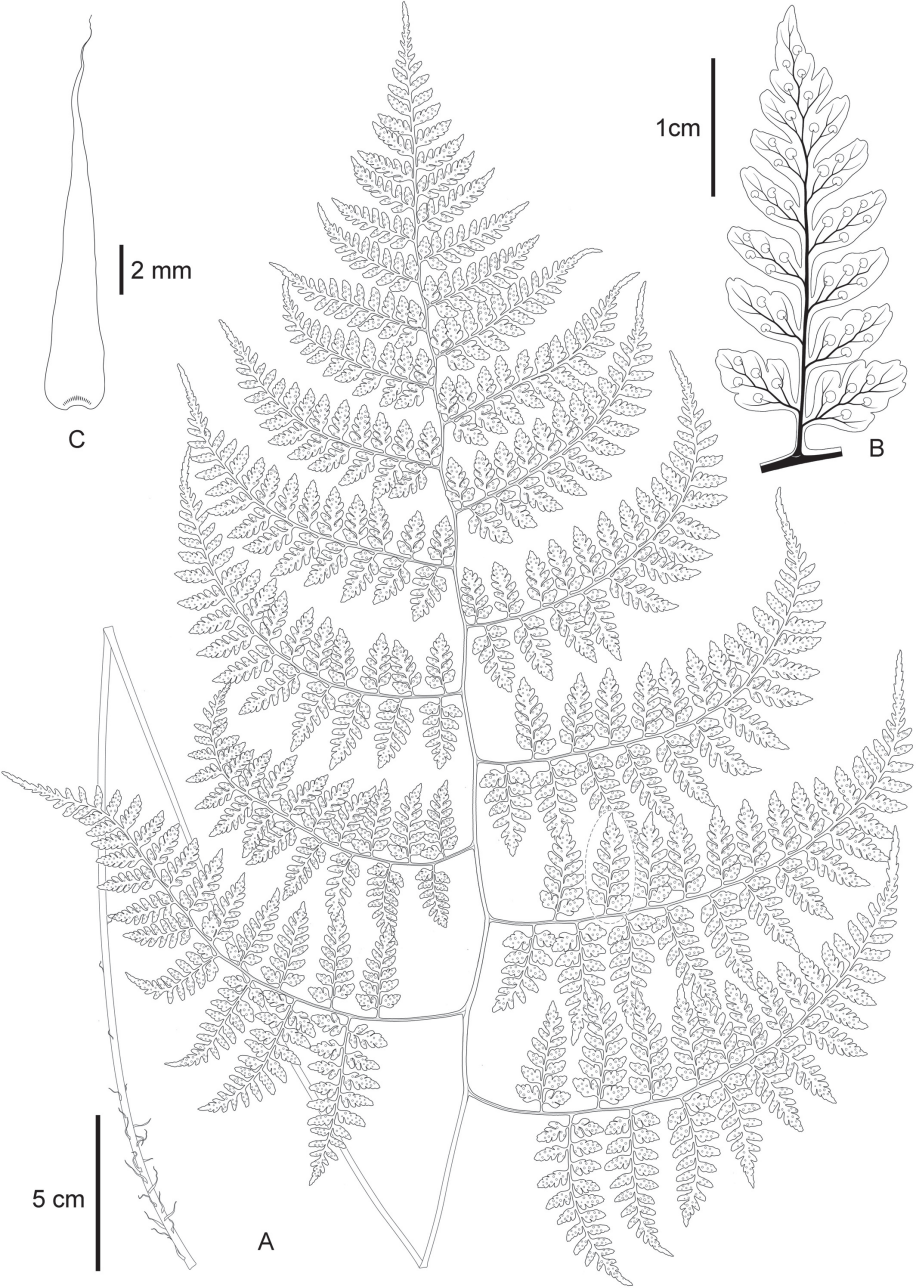
Dryopteris×subdiffracta**A** habit **B** portion of costa with pinnule showing veins and sori **C** scale from stipe base. Illustrated by Hong-Jin Wei.

**Figure 5.**
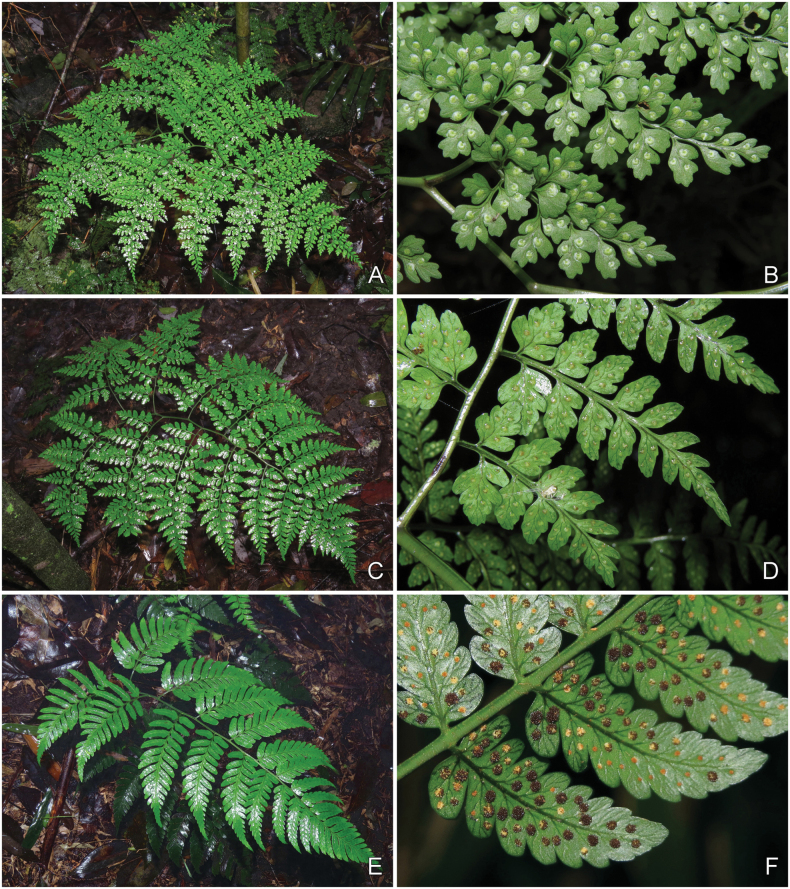
*Dryopterisdiffracta* (top), *D.×subdiffracta* (middle) and *D.polita* (bottom) **A**, **C**, **E** habit **B** portion of rachis with portion of upper pinna **D** portion of rachis with portion of middle pinna **F** portion of middle pinna. Photographed by Hong-Jin Wei.

##### Geographical distribution and ecology.

**Figure 6.**
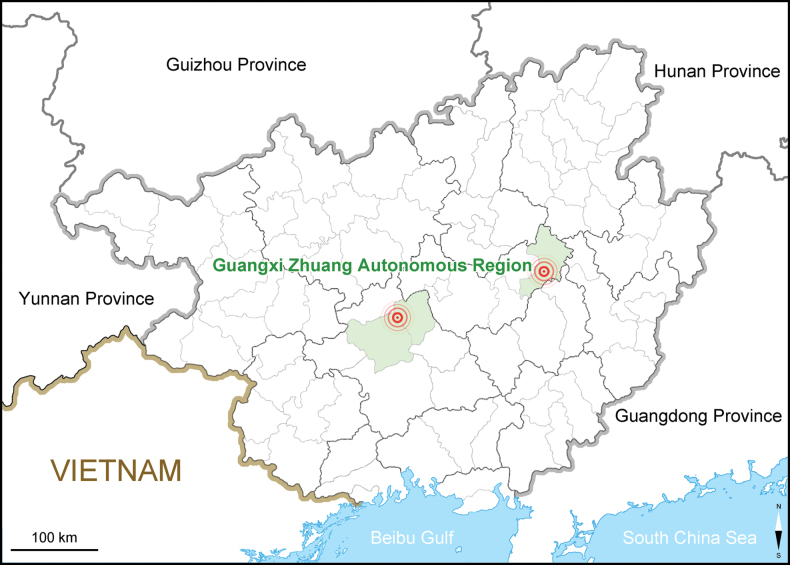
Geographical distributions of Dryopteris×subdiffracta (centers of rings) in Guangxi Zhuang Autonomous Region, China.

Dryopteris×subdiffracta was found in Jinxiu, Shangling and Wumin County, Guangxi Zhuang Autonomous Region, South China (Fig. 6), always growing together with *D.diffracta* and *D.polita* in broad-leaved forests at the elevation of 800–1200 m.

We noticed that two specimens of D.×subreflexipinna (TAI270081, TAI270101) collected from Taiwan are consistent with the characteristics of the new hybrid. There is a strong possibility that these two specimens were collected in the area where *D.diffracta* coexisted with *D.polita. Dryopteris*×subdiffracta might be distributed to Taiwan.

##### Etymology.

The specific name is derived from the Latin prefix sub- (close to), and diffracta, epitheton of a species, referring the new species is closely related to *Dryopterisdiffracta*.

##### Chinese name.

曲轴鳞毛蕨 (qū zhóu lín máo jué).

##### Paratypes.

China • Guangxi: Same place as the holotype, 1100–1200 m, 9 May 2018, *She-Lang Jin, Jing Liu, Qi-MingTang & Xu Yan JSL6341* (CSH!), *Jing Liu, She-Lang Jin, Qi-MingTang & Xu Yan DYS426, DYS427, DYS428* (IBK!) • ibid., 15 Aug. 2023, *Zheng-Yu Zuo Zuo5589* (KUN!) • ibid., 2 Mar. 2024, *Ming-jin Wei & Yu-jin Wei JSL9482* (CSH!).

##### Additional specimens examined.

China • Guangxi: Wumin County, Mt. Damingshan, Gannanhe (Ganlanhe), elev. 800–1000 m, 14 Sep. 1991, *Hou-Gao Zhou & Hua Li 2769* (IBK!) • Shangling County, Mt. Damingshan, Ganlanhe, elev. 900 m,16 Jul. 2011, *Lei Wu & She-Lang Jin D2760* (IBK!).

## Supplementary Material

XML Treatment for Dryopteris × subdiffracta

## ﻿Additional information

### Author ORCIDs

Hong-Jin Wei https://orcid.org/0000-0002-3679-6683

Zheng-Yu Zuo https://orcid.org/0000-0002-8334-6132

### Data availability

All of the data that support the findings of this study are available in the main text.
